# Supplementary material for: Flagellin is essential for initial attachment to mucosal surfaces by Clostridioides difficile
Source: Microbiol Spectr. 2023 Oct 12;11(6):e02120-23. doi: 10.1128/spectrum.02120-23 (PMC10714722; doi:10.1128/spectrum.02120-23)
Supplement: Supplemental material — Fig. S1 to S4 and Table S1. [file spectrum.02120-23-s0001.pdf]

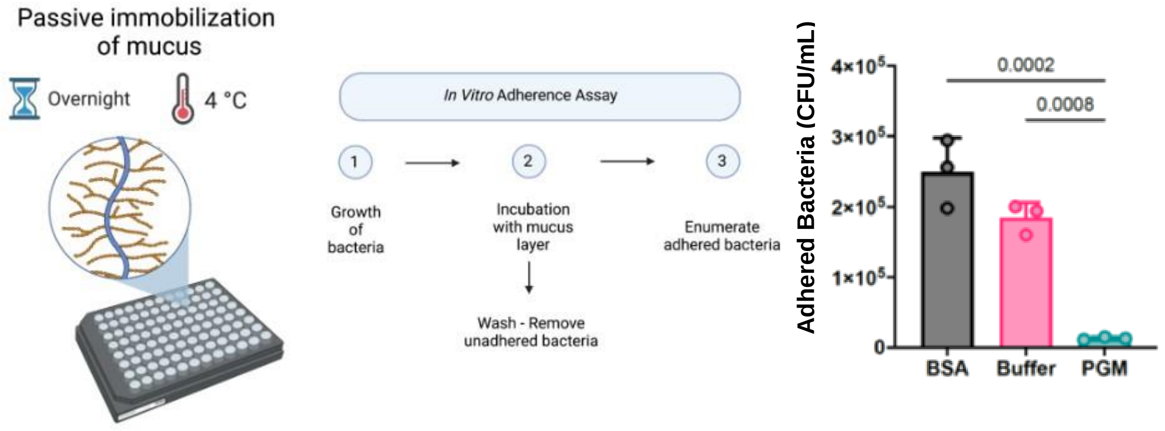

Supplementary Figure 1: Immobilization of mucus onto high-binding plates. Porcine Gastric Mucus (Sigma) was attached to high-binding plates overnight at 4C and blocked with bovine serum albumin (Fisher) Adherence by Cd R20291 was measured after incubation for one hour at 37C by enumeration of colony forming units after washing and removal by trypsin (See Methods). Greater adherence was measured to wells not coated with mucins (with or without blocking with BSA), suggesting that non-specific adhesion to plastic surfaces by *C. difficile* is potentially equal in strength to many biomolecular interactions. Significant differences were calculated using ANOVA with corrections for unequal variance.

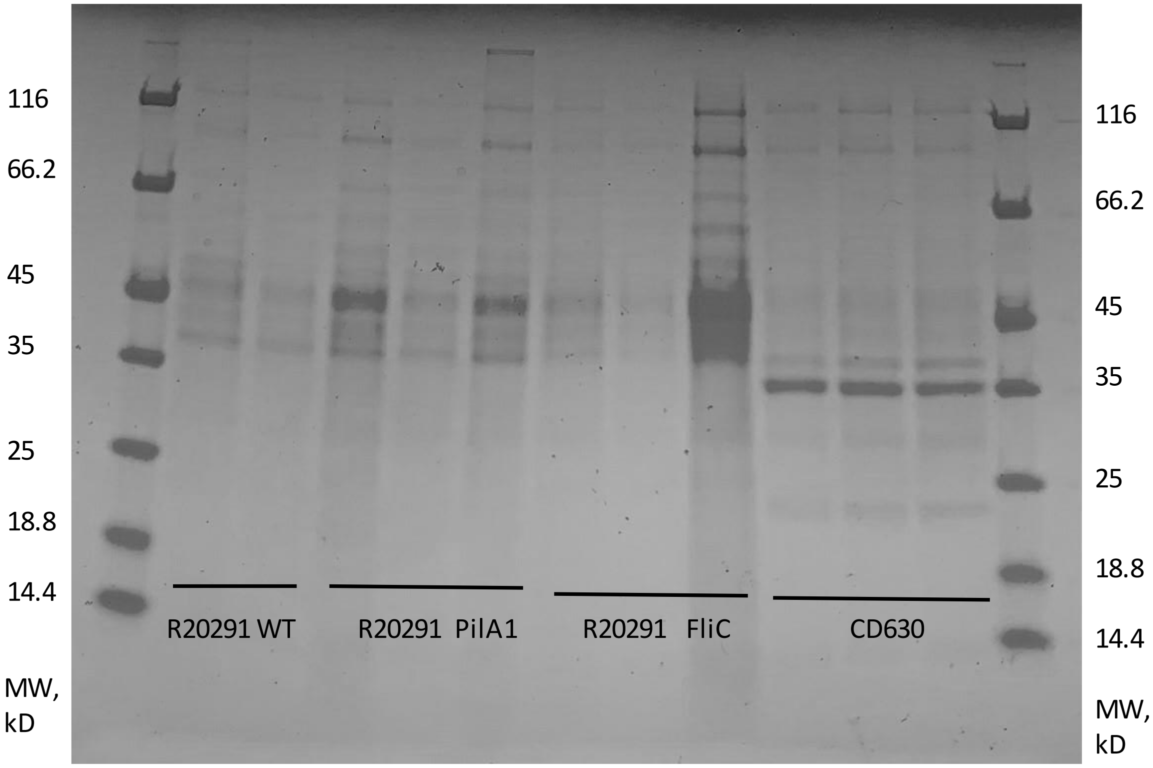

Supplementary Figure 2: Isolation of *C. difficile* surface proteins from cells through shearing. A) After 24 hours of growth in BHIS broth, flagella were sheared from the surface of *C. difficile* R20291 and mutants through vortexing (10 seconds) alternated with incubation on ice (20 seconds) for a total of 60 seconds of vortexing in anaerobic sealed tubes. The suspension was centrifuged at 4000 x g for 10 minutes to pellet the cells. The pellets were discarded and ammonium sulfate was then added to the supernatant at 30% and they were stirred overnight at 4C. These suspensions were centrifuged at 20,000 x g for 30 minutes to pellet any precipitated proteins. Those pellets were resuspended in PBS at run on SDS page shown above.

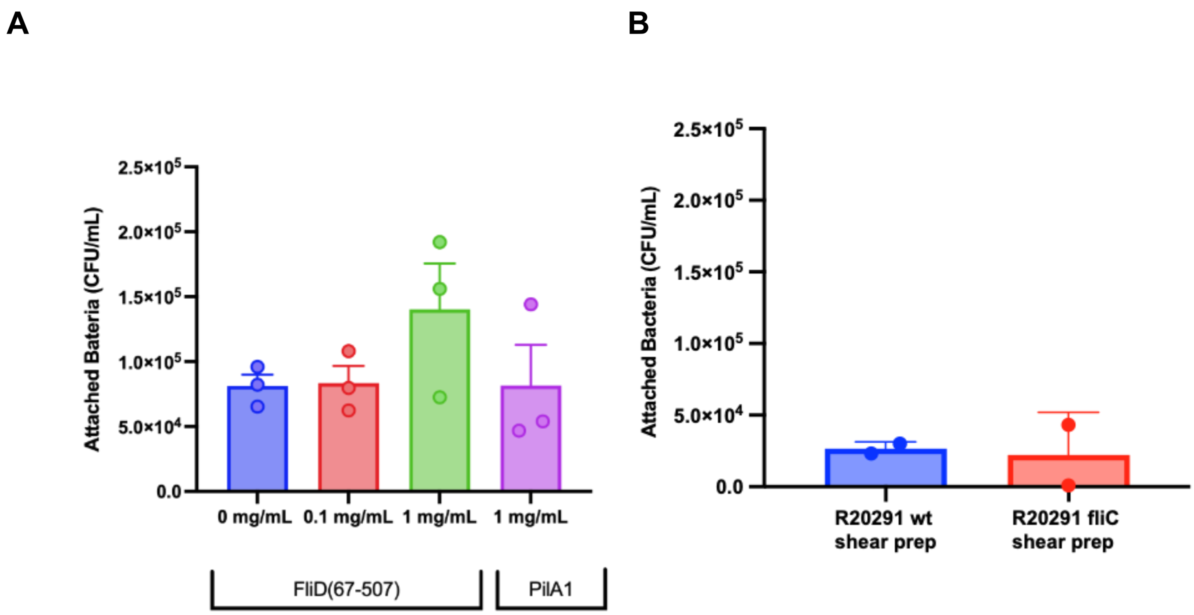

Supplemental Figure 3: Competitive inhibition of Cd FliD and isolated flagella in mucosal binding assays. Mucosal binding experiments with Cd R20291 binding to porcine gastric mucin were performed as previously described with the addition of either A) Cd FliD from recombinant expression in *E. coli* or B) surface proteins (including flagella when present) sheared from cell surface as described above at approximately 1 mg/ml. No differences between groups were significant.

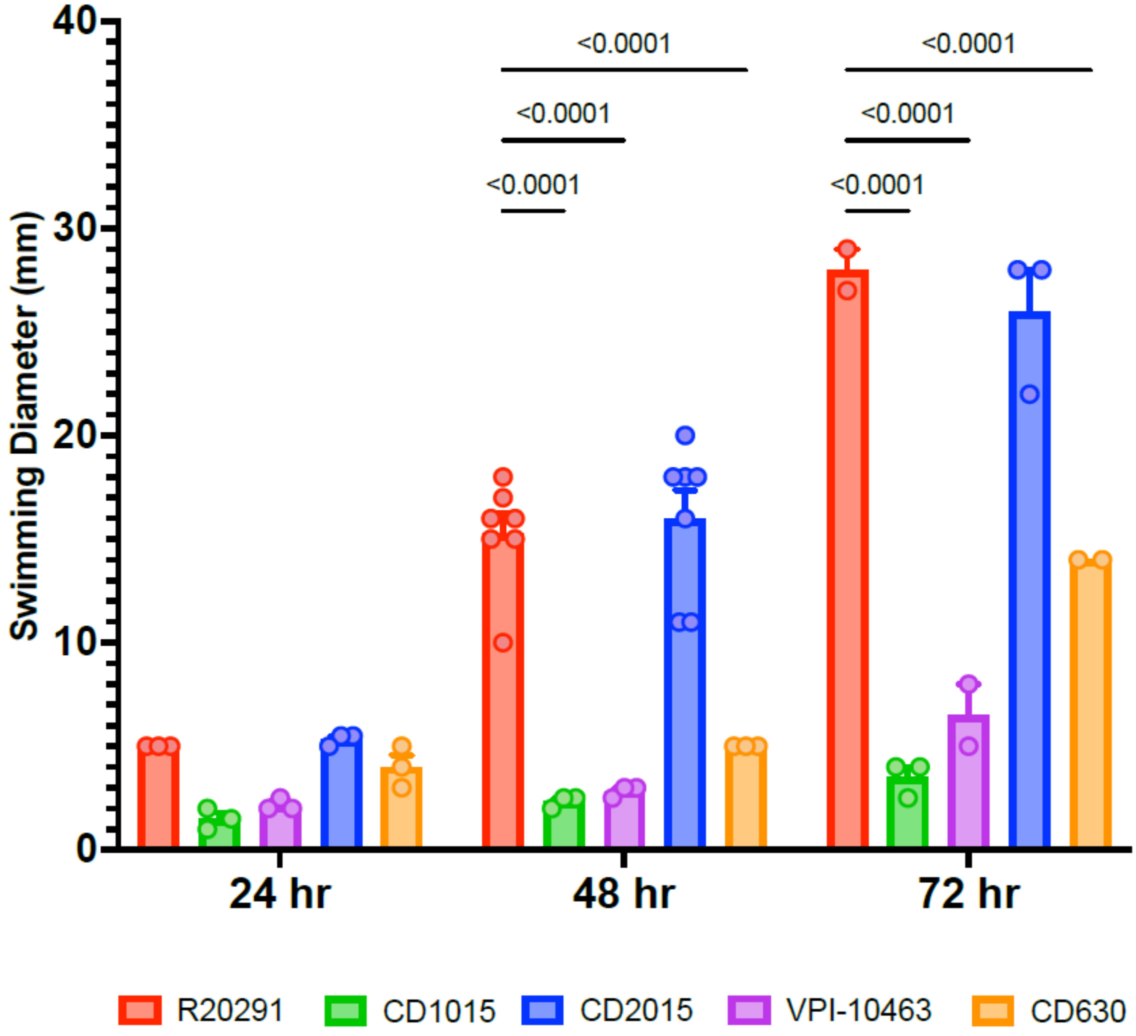

Supplemental Figure 4: Swimming motility over time in 0.3% agar, measured as the diameter of the bacterial expansion after 24, 48 and 72 hours. Using ANOVA with corrections for unequal variance, all differences between groups were calculated with only significant differences shown.

***C. difficile* strain**	**Reference**	**Description**
**CD37**	25,26	Non-toxigenic ribotype 009 clade 1 isolate
**CD630**	19, 20	Virulent ribotype 012 clade 1 clinical isolate.
**R20291**	19, 20	Hypervirulent ribotype 027 clade 2 clinical isolate .
**R20291 *pilA***	19, 20	R20291 *pilA* gene interruption mutant produced using the ClosTron system.
**R20291 *fliC***	19, 20	R20291 *fliC* gene interruption mutant produced using the ClosTron system.
**R20291 *pilA* p84151-*pilA***	19, 20	R20291 *pilA* complemented strain. Intact *pilA* gene cloned into the p80000 shuttle vector p84151 and conjugated into gene interrupted mutant strain.
**R20291 *fliC* p84151-*fliC***	19, 20	R20291 *fliC* complemented strain. Intact *fliC* gene cloned into the p80000 shuttle vector p84151 and conjugated into gene interrupted mutant strain.
**CD2015**	22, 23	Hypervirulent ribotype 027 clade 2 clinical isolate.
**CD1015**	22, 23	Ribotype 078 clade 5 clinical isolate.
**CD2048**	22	ribotype 053 clade 1 clinical isolate
**CD1014**	22	ribotype 014 clade 1 clinical isolate
**VPI-10463**	24	High toxin producer ribotype 03 clinical isolate.

Supplemental Table 1:strains and mutants
